# β-Glucans: Multi-Functional Modulator of Wound Healing

**DOI:** 10.3390/molecules23040806

**Published:** 2018-04-01

**Authors:** Juraj Majtan, Milos Jesenak

**Affiliations:** 1 Institute of Molecular Biology, Slovak Academy of Sciences, Dubravska Cesta 21, 845 51 Bratislava, Slovakia; 2 Department of Paediatrics, Jessenius Faculty of Medicine, Comenius University in Bratislava, Kollarova 2, 036 59 Martin, Slovakia; jesenak@gmail.com

**Keywords:** polysaccharide, natural product, immunomodulator, wound repair

## Abstract

β-glucans are derived from a variety of sources including yeast, grain and fungus and belong to the class of drugs known as biological response modifiers. They possess a broad spectrum of biological activities that enhance immunity in humans. One promising area for β-glucans’ application is dermatology, including wound care. Topical applications of β-glucans are increasing, especially due to their pluripotent properties. Macrophages, keratinocytes and fibroblasts are considered the main target cells of β-glucans during wound healing. β-glucans enhance wound repair by increasing the infiltration of macrophages, which stimulates tissue granulation, collagen deposition and reepithelialization. β-glucan wound dressings represent a suitable wound healing agent, with great stability and resistance to wound proteases. This review summarizes the current knowledge and progress made on characterizing β-glucans’ wound healing properties in vitro and in vivo and their safety and efficacy in managing non-healing wounds or other chronic dermatological conditions and diseases.

## 1. Introduction

β-Glucans, which are glucose polymers, from a variety of sources including yeast, grain and fungus, belong to the class of drugs known as biological response modifiers [1]. Numerous studies have shown that β-d-glucans, either particulate or soluble, enhance immune functions with anti-infective, antitumor and immunomodulatory activity [2,3,4,5,6]. Additionally, they have extensive treatment applications in healthcare, not only for humans, but also invertebrates, rodents, fish and domestic farm animals due to their marked capability of modulating the immune system (reviewed in [7]). All β-glucans are glucose polymers linked by 1,3; 1,4 or 1,6 β-glycosidic bonds and differ from each other by their length and branching structure. The biological actions of the different β-glucans vary according to their molecular structure, solubility and the conformation of each polymer [8].

The mechanism of β-glucan action in the organism is mediated by several receptors, especially the Dectin-1 receptor, Toll-like receptors (TLR-2, 4, 6), complement receptor 3 (CR3), scavenger receptor and lactosylceramide [9]. The most important is the Dectin-1 receptor, which is highly expressed in many immunocompetent cells such as dendritic cells (DC), neutrophils, eosinophils, macrophages, monocytes, several T lymphocytes and also in cutaneous cells (keratinocytes and fibroblasts). After binding to the Dectin-1 receptor, β-glucan stimulates the production of many cytokines or activates other immune and non-immune reaction mechanisms [10].

One promising area of β-glucan application is dermatology, including wound care. Topical application of β-glucans is increasing, since their pluripotent activity (antioxidant, anti-inflammatory and regenerative effects, immunomodulation, radioprotection, moisturization and rejuvenation) might help as a complementary therapy in managing various skin diseases and conditions [11,12,13]. In addition, some β-glucans also possess anti-infective properties and exhibit potential antibacterial activity against a broad spectrum of Gram-positive and Gram-negative bacteria [14]. β-Glucans may therefore represent a suitable wound healing agent with great stability and a broad range of biological activities.

**Figure 1:**
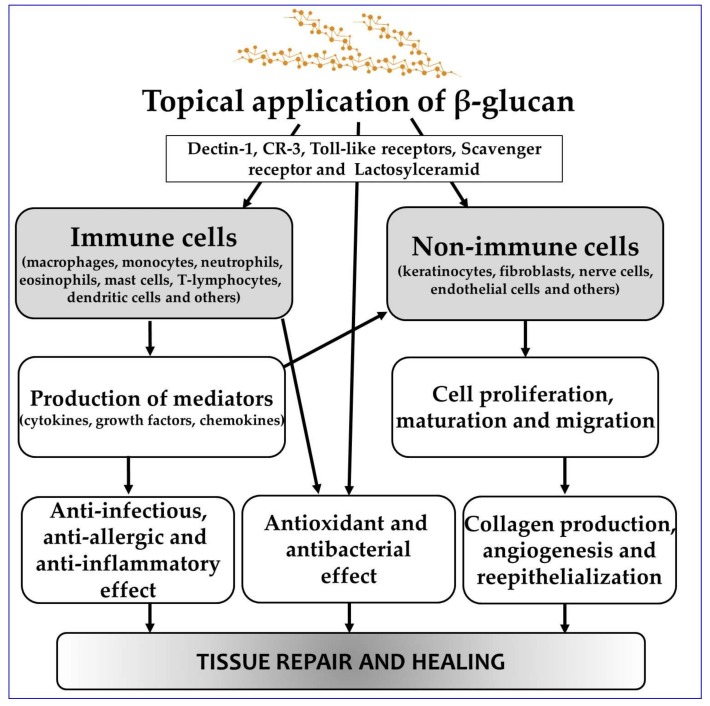
Schematic depiction of β-glucan pluripotent mechanisms in wound healing.

Wound healing is a complex process involving various cellular and extracellular matrix components and cells (keratinocytes, fibroblasts, endothelial cells, mast cells, nerve cells and leucocyte subtypes) that participate differently in the three overlapping phases (inflammation, cell proliferation and tissue remodeling) [15]. As mentioned above, the recognition of and response to β-glucans are mediated primarily by cell surface receptors, including those from immunocytes and cutaneous cells. In terms of β-glucan immunostimulatory activity regarding wound healing, two modes of action are possible. One manifests by indirect activation through various cytokines of macrophages and the other by direct influence on keratinocytes and fibroblasts. When released, the range of growth factors from activated macrophages supports cellular proliferation, angiogenesis, reepithelialization and an increase in wound tensile strength [16]. A schematic depiction of the β-glucan pluripotent mechanisms of action is shown in Figure 1.

This review summarizes the current knowledge and progress made on characterizing β-glucan wound healing properties in vitro and in vivo and their safety and efficacy in managing non-healing wounds and burns.

## 2. Wound Healing Activity of β-Glucans: In Vitro Experiments

β-Glucans have been shown to possess interesting biological properties, including anti-inflammatory and immunomodulatory activity that could be effectively employed in the wound healing process. It has been proposed that β-glucans accelerate the healing process in both chronic and acute wounds [17]. Chronic wounds are characterized by a prolonged inflammatory phase where macrophages in granulation tissue, after stimulation with β-glucans, act as a source of growth factors and inflammatory cytokines (IL-6, IL-1β and TNFα), this pro-inflammatory event being mediated by the Dectin-1 receptor [18]. In another experiment, it was verified that macrophages can also stimulate other cells such as keratinocytes and fibroblasts that reepithelialize the wound and create granulation tissue [19,20]. 

β-Glucans also exhibited in vitro antimicrobial activity [21,22,23], directly against a broad range of bacterial species, including *Escherichia coli*, *Pseudomonas aeruginosa* and *Staphylococcus aureus*, or indirectly by enhancing macrophage phagocytic activity and resistance to bacterial challenge [24,25]. In another study, β-glucan from oats also showed antimicrobial effects against *Escherichia coli* and *Bacillus subtilis* [21]. Native β-glucans were able to partially (35% inhibition) inhibit the growth of these bacteria, while the cationic β-glucan caused 80% inhibition in both types of bacteria, indicating that β-glucan amination enhanced the antimicrobial effects. The antibacterial action mechanism of aminated oat β-glucans can be explained by the interaction of polycations with the negatively-charged bacterial surface, which alters membrane permeability and thereby inhibits growth. Thus, β-glucans, particularly derivatized ones, may inhibit the bacterial growth of wound pathogens and prevent biofilm formation in chronic wounds [21].

The in vitro wound healing properties of β-glucans have been well described, and most studies have been performed using cutaneous cells (fibroblasts and keratinocytes). Overall, β-glucans with different molecular weights, origins and physical and chemical characteristics were shown to be potent inducers of wound closure in vitro, significantly affecting the migration and proliferation of cells involved in wound repair. The major properties of β-glucan wound healing are summarized in Table 1.

## 3. Wound Healing Activity of β-Glucans: Animal Studies

The findings of laboratory studies have provided compelling evidence that β-glucans can accelerate the wound healing process and inhibit the growth of pathogenic bacteria. Many research groups have therefore started paying attention to the topical application of β-glucans and explaining its wound healing contribution by stimulating the tissue regeneration, collagen deposition and reepithelialization and increasing wound tensile strength. Animal studies have been performed with different model organisms such as rats, mice and aquatic organisms (carp and trout) (see Table 2). 

To evaluate the role of β-glucans in wound healing pre-clinical studies, only in vivo studies indexed in the Scopus and PubMed databases were considered. Based on this approach, 17 were identified and included in Table 2: eight of the studies involved a mouse wound model; seven used a rat wound model; and two were conducted using a carp and trout model.

Overall, β-glucan preparations or membranes/hydrogels incorporated with β-glucans with different solubility and origin were shown to be effective wound healing agents in all animal pre-clinical studies. In some cases, β-glucan was administrated both topically and systematically. The majority of the studies investigated the wound healing effect of β-glucan in an excised sterile wound model, and only one study [40] demonstrated the healing potential of β-glucan in an infected wound model. Contrary to some in vitro studies, in which a direct antibacterial effect of β-glucan was demonstrated, no evidence of the β-glucan antibacterial effect in in vivo studies is available.

## 4. Clinical Evidence for the Use of β-Glucans in the Management of Human Non-Healing Wounds and Burns

Despite the abundance of published data on in vitro and in vivo β-glucan effects in wound healing, to date, only a few human clinical studies have been conducted utilizing β-glucans in wound care management [54,55,56,57]. According to the web resource www.clinicaltrials.gov (date: 1 October 2018; terms: wound and glucan; conditions: all studies; country: all countries), two human randomized clinical studies with an estimated number of participants of 80 per trial are currently in progress focusing on the treatment of diabetic foot ulcers and venous leg ulcers using β-glucan-based products.

The first human study, in which the authors evaluated the effectiveness of a β-glucan cream (containing pleuran from *Pleurotus ostreatus*) as a topical treatment for venous ulcers, was published in the proceedings of a scientific meeting [58], followed by another study published in 2012 [54]. In this non-randomized trial with intragroup comparisons over time, water-insoluble (1→3)-β-glucan isolated from *Saccharomyces cerevisiae* was applied in the form of a cream with a final concentration of 3% (*w*/*w*) directly onto the ulcer bed of 12 patients [54]. This procedure was performed daily for up to 90 days. The authors documented that the average percentage of reduction of an ulcer was 11.3% after 30 days of treatment and 55.23% after 90 days. Insoluble β-glucan has been shown to enhance venous ulcer healing and increase epithelial hyperplasia, as well as increase plasmocyte and fibroblast proliferation.

In fact, four human clinical studies involving less than 90 participants provided evidence that β-glucan can accelerate the wound healing of chronic wounds (Table 3). Two of these clinical studies used water-insoluble β-glucan [54,57], and two other studies used water-soluble β-glucan [55,56]. Only one clinical study [55] represented a double blind, placebo-controlled trial. 

In the case of burn injuries, a β-glucan collagen matrix was used as a primary wound dressing to treat pediatric partial thickness burns in 43 patients [59]. The β-glucan dressing reduced the pain, improved wound healing and had excellent cosmetic results in 79% of patients. The authors did not document any adverse effects during β-glucan treatment.

The treatment of chronic wounds is protracted and intensive and associated with high costs. Various approaches have been developed to treat chronic wounds, including topical wound-care therapies. The major arguments for employing natural products are the low cost and absence of antimicrobial resistance risk compared to conventional wound-care products. In a very recent study [60], Cutting evaluated the economic benefits of a β-glucan gel in the treatment of diabetic foot ulcers using a Markov cohort simulation model. The analysis revealed cost savings over an annual budget cycle of £GBP 503.00 per patient. Additionally, β-glucan gel was able to heal 94% of wounds compared to 78% from the ones of the standard care. 

Generally, all the conducted human clinical studies suggest that β-glucan is an effective, safe, well-tolerated and economic wound dressing for the treatment of non-healing wounds and burns.

## 5. Sterilization of β-Glucans and Their Impact on β-Glucan Bioactivity

Today, β-glucans represent effective topical agents for the treatment of chronic wounds and burns. However, licensed wound care products containing β-glucan must undergo a sterilization process. In fact, the most therapeutic compositions for clinical testing in humans are sterilized, and in many cases, sterilization is required to meet stringent regulations. Sterilization of β-glucan-based products is vital due to the presence of potential harmful contaminants, such as vegetative bacteria and microbial spores. However, some traditional sterilization techniques such as ethylene oxide and heat may have negative effects on chemical and physical properties and may thus alter β-glucan’s biological properties [61]. Some sterilization methods such as filtration are also time consuming and difficult to perform on polymeric viscous solutions. Therefore, some other means of sterilization have to be adopted, such as gamma irradiation.

### Gamma Irradiation

Gamma irradiation, as well as X-ray and an electron beam may cause physical and chemical changes in the β-glucan structure. These techniques are being recognized as effective tools in lowering the molecular weight of β-glucans to enhance their solubility and permeability into the cells and to lessen viscosity. Gamma irradiation is a relatively simple method and leads to an increase in the functionality of β-glucans [62]. Several studies have investigated the effect of irradiating β-glucans on their biological and pharmacological properties [63,64,65,66]. Different gamma irradiation doses were used, starting from 2 up to 75 kGy. A significant decrease in the molecular weight of non-irradiated β-glucans has already been documented to occur at a low irradiation dose (8 kGy) [64]. Among the studied irradiation doses, 50 kGy proved to be an effective dose, allowing the maintenance of excellent antioxidant and functional properties. Further, the physical properties (e.g., gel fraction, compressive strength) of irradiated β-glucans were enhanced by increasing the irradiation dose [41]. Taken together, irradiated β-glucan exhibits: (a) a higher antimicrobial activity compared to the native samples; (b) an enhanced antioxidant, antitumor and antiproliferative activity; and finally, (c) an enhanced response in the immune system, suggesting that smaller molecules of irradiated β-glucans have a higher chance of binding to a receptor.

Gamma irradiation has successfully been used in preparing fabricated β-glucan hydrogels for wound dressings [41]. In this study, Gwon et al. verified that the irradiated hydrogels stimulated wound repair in an animal wound model; therefore, irradiated β-glucan may be considered a good tissue regeneration agent with strengthened wound healing properties.

## 6. Conclusions

In vivo studies and human clinical trials have provided compelling evidence that β-glucan preparations including hydrogels and nanofibers with incorporated β-glucan molecules promote moist wound healing and repair mainly due to the activation of the immune and cutaneous cells. β-glucans can induce the proliferation and migration of keratinocytes and fibroblasts through specific receptors such as Dectin-1, CR3 or TLRs. The direct antibacterial properties of β-glucans remain controversial as they have not been demonstrated in vivo. However, it was proposed that activation of wound macrophages and antigen-presenting cells by β-glucan represents a potential mechanism in eradicating bacterial load from chronic wounds.

Besides therapeutic benefits, treating chronic wounds with β-glucan hydrogels can help with reducing healing time and the total costs per treatment, but additional human clinical studies are needed to prove the efficacy of β-glucan-based wound dressings in treating a broad spectrum of chronic wounds and burns.

## Figures and Tables

**Table 1:** β-Glucan in vitro wound healing properties.

Type of β-Glucan/Source	Structure of β-Glucan	Target Cell Type	Actions	Ref.
W-S Curdlan, (7%)/*Agrobacterium* sp.	Linear (1,3;1,6) β-glucan	Swiss-3T3 fibroblast cells	Fibroblasts exposed to a curdlan/polyvinyl alcohol (PVA) blend showed significantly higher spreading rates in the scratch wound assay after 24 h when compared to control and pure PVA.	[26]
W-INS (2%)/*Saccharomyces cerevisiae*	Linear (1,3) β-glucan	3T3 fibroblast cells, HaCaTs	β-glucan-based nanofibrous membranes promoted the adhesion and proliferation of fibroblasts and keratinocytes.	[27]
W-S (2.5%)/*Aureobasidium pullulans*	Branched (1,3;1,6) β-glucan	Human fetal dermal fibroblast cell line FW20-2, primary human dermal fibroblasts	β-glucan consistently stimulated dermal fibroblast proliferation and migration and modulated the effect of transforming growth factor-β_1_. β-glucan l did not affect the procollagen production from fibroblasts.	[28]
W-S (0.75%)/*Bradyrhizobium japonicum*	Cyclic (1,3;1,6) β-glucan	Swiss-3T3 fibroblast cells	Cyclic β-glucan incorporated in hydrogels increased hydrogel porosity and enhanced cell attachment, proliferation and migration activity.	[29]
W-S Curdlan, (10 μg/mL)/*Alcaligenes faecalis*	Linear (1,3) β-glucan	Primary human keratinocytes	Curdlan enhanced migration, proliferation and wound closure of primary human keratinocytes in a Dectin-1 concentration-dependent manner.	[30]
W-S Laminarin (0.2 mg/mL)/*Laminaria* sp. W-INS Paramylon (0.2 mg/mL)/*Euglena gracilis*	Linear (1,3) β-glucan with (1,6)-linked β-glucosyl Linear (1,3) β-glucan	Immortalized human corneal epithelial cells	Both β-glucan types promoted epithelial migration in a concentration-dependent manner. This effect was enhanced when β-glucan was conjugated with hyaluronic acid to increase solubility.	[31]
W-INS (1 mg/mL)/*Saccharomyces cerevisiae*	Branched (1,3;1,6) β-glucan	Human and mouse wound macrophages	β-glucan potently induced TNF-α production in wound macrophages via activation of the Dectin-1 and TLR2 signaling pathways. Interestingly, it also induced phosphorylation of the macrophage-colony stimulating factor.	[32]
W-INS, W-S (1 μg/mL)/various sources	Linear (1,3;1,4) β-glucanBranched (1,3;1,6) β-glucan Linear (1,3) β-glucan	HaCaTs, 3T3-Li preadipocytes	β-glucans of different origin, solubility and purity were tested for inhibition of adipogenic differentiation, wound healing and skin irritation. Significant differences in efficacy between β-glucan preparations were shown. The most active was yeast-derived insoluble β-glucan.	[33]
W-S (1 mg/mL)/*Aureobasidium pullulans*	Branched (1,3;1,6) β-glucan	Human dermal fibroblast, adipose tissue-derived stem cells	β-glucan enhanced cellular response, proliferation and migration of both human dermal fibroblasts and adipose tissue-derived stem cells.	[34]
W-INS Pleuran, (0.2 mg/mL)/*Pleurotus ostreatus*	Branched (1,3;1,6) β-glucan	Primary human keratinocytes	β-glucan induced the production of matrix metalloproteinase-9 from human keratinocytes.	[35]
W-S (0.5–1 mg/mL)/*Aureobasidium* sp.	Branched (1,3;1,6) β-glucan	Mouse macrophage cell line RAW264.7, human dermal fibroblasts	β-glucan accelerated wound healing by enhancing fibroblast migration and collagen synthesis via glucan receptors on fibroblasts, as well as by activating macrophages to release more TNF-α.	[20]
W-S (0.5 and 5 mg/mL)/*Lentinula edodes*	Linear (1,3) β-glucan with (1,6)-linked- β-glucosyl	Mouse connective tissue L-929 cells	β-glucan treatment of L-929 cells resulted in a dose-dependent increase in cell proliferation. No significant difference in the migration speed between the glucan-treated and non-treated cell was documented.	[36]
W-S (1 μg/mL)/*Saccharomyces cerevisiae*	Branched (1,3;1,6) β-glucan	Human dermal fibroblasts	Derivatized β-glucan increased nuclear factor-1 binding capacity and stimulated collagen biosynthesis in human dermal fibroblasts.	[19]
W-S (1 μg/mL)/*Saccharomyces cerevisiae*	Branched (1,3;1,6) β-glucan	Normal human dermal fibroblasts	Derivatized β-glucan stimulated the expression of cytokine and procollagen genes through the transcription factors activator protein-1 and specificity protein-1 in normal human dermal fibroblasts. It also stimulated expression of various growth factors participating in wound repair.	[37]
W-S (1 mg/mL)/*Saccharomyces cerevisiae*	Branched (1,3;1,6) β-glucan	Normal human dermal fibroblasts	β-glucan stimulated fibroblast NF-kB nuclear binding activity and interleukin 6 gene expression in a time-dependent manner. It can directly modulate the functional activity of human dermal fibroblasts. At least two binding sites for β-glucan on fibroblasts were identified.	[38]
W-S (0.01%)/*Saccharomyces cerevisiae*	Branched (1,3;1,6) β-glucan	Porcine keratinocytes	Carboxymethyl β-glucan at a concentration of 0.005 and 0.01% stimulated keratinocytes proliferation within 120 h of incubation in culture media.	[39]

Note: W-INS, water-insoluble; W-S, water-soluble.

**Table 2:** β-Glucan in vivo wound healing properties.

Type of β-Glucan/Source	Structure of β-glucan	Animal Model	Actions	Ref.
W-INS (4%)/*Schizophyllum commune*	Branched (1,3;1,6) β-glucan	Sprague-Dawley rat	Hydrogels incorporating β-glucan and sterilized with gamma radiation accelerated wound repair while releasing of β-glucan molecules from the hydrogel.	[41]
W-INS (2%)/*Saccharomyces cerevisiae*	Linear (1,3) β-glucan	Kunming mouse	β-glucan-based nanofibrous membranes significantly reduced wound size compared to control groups. On Day 14, 83% wound size reduction was observed (26% in the control group). All wounds were completely covered with epidermis by Day 14, but the β-glucan group exhibited substantially more epithelialization.	[27]
W-S (0.75%)/*Bradyrhizobium japonicum*	Cyclic (1,3) β-glucan	Wistar rat	Nanofibrous asymmetric membranes containing β-glucan significantly accelerated wound healing by promoting reepithelialization, tissue remodeling and collagen deposition. Collagen deposition was more organized than in the control group. The levels of pro-inflammatory cytokines at Day 8 were significantly lower in the β-glucan group compared to the control group.	[29]
W-S (0.1 mg/L)/*Saccharomyces cerevisiae*	Branched (1,3;1,6) β-glucan	Rainbow trout	Wound healing progressed, at least partially, due to a low temperature of about 8.5 °C: β-glucan was shown to have a very limited effect on wound healing in trout, when in a bath. Complete healing was achieved only by Day 100.	[42]
W-S (2.5%)/*Aureobasidium pullulans*	Branched (1,3;1,6) β-glucan	Diabetic C57BL/KsJ-db/db mouse	Treatment with β-glucan caused a significant decrease in wound size compared to the vehicle control (distilled water). Increased inflammatory cells in the granulation tissues of wound areas were also significantly less compared to the vehicle control, showing increased numbers of micro-vessels and fibroblasts, as well as reepithelialization.	[43]
W-S Laminarin (0.2 mg/mL)/*Laminaria* sp.	Linear (1,3) β-glucan with (1,6)-linked β-glucosyl Linear (1,3) β-glucan	Sprague-Dawley rat	β-glucan and β-glucan conjugated with hyaluronic acid suppressed acute inflammatory reactions in the rat corneal alkali burn model. Decreased corneal edema and less polymorphonuclear leukocytes infiltrates were observed in the β-glucan-treated wound.	[31]
W-S MacroGrad, (0.1 μg/mL)/*Saccharomyces cerevisiae*	Branched (1,3;1,6) β-glucan	*Cyprinus carpio* L (carp)	β-glucan promoted the wound healing process in common carp compared to the group treated with 6.3 kDa oat fiber and the control fish group. The positive effect of the β-glucan preparation was related to the high branching levels due to fish being bathed in β-glucan-supplemented water, showing a higher wound closure ratio compared to a 6.3-kDa-supplemented bath.	[44]
W-S (1 mg/mL)/*Sparassis crispa*	Branched (1,3;1,6) β-glucan	ICR mouse	Fungal β-glucan significantly increased the rate of wound healing due to, at least partially, local stimulation of collagen synthesis.	[45]
W-S (2.5%)/*Aureobasidium pullulans*	Branched (1,3;1,6) β-glucan	ICR nu/nu mouse	β-glucan promoted wound healing of full-thickness wounds infected by *S. aureus*, *S. pyogenes* and *P. aeruginosa*. β-glucan did not show any direct antibacterial activity, though it was able to heal the wounds.	[40]
W-S (50%)/*Aureobasidium pullulans*	Branched (1,3;1,6) β-glucan	BALB/c nude mouse	Poly-(lactic-co-glycolic acid) (PLGA), a constituent of the membranes containing 50% β-glucan, enhanced the wound’s interaction with the surrounding cells, proliferation and angiogenesis compared to the ones from the membranes without PLGA. A PLGA membrane with incorporated β-glucan may be applied as a skin substitute to accelerate wound healing.	[46]
W-S Curdlan, (11.1 mg/mL)/*Alcaligenes faecalis*	Linear (1,3) β-glucan	Diabetic C57Bl/KsBom-*db/db*	β-glucan exerted its primary effects in the early phase of wound healing and was coherent with an effect on macrophage functions in the wound. A higher dosage frequency showed a significant improvement in wound closure compared to a low dosage frequency.	[47]
W-S (1%)/*Aureobasidium pullulans*	Branched (1,3;1,6) β-glucan	ddY mouse	A β-glucan and chitosan complex was used for wound healing. The complex sheet did not dissolve during the application period, did not adhere to the wound and was easy to remove. The complex accelerated wound repair by activating macrophages and cytokines’ release and accelerated reepithelialization of the skin wound.	[48]
W-INS Imuneks, (5% cream or 50 mg/kg)/*Saccharomyces cerevisiae*	Branched (1,3;1,6) β-glucan	Wistar rat	Systemic and local administration of β-glucan stimulated wound contraction, increased incision tensile strength and improved epithelialization. Systemic administration was more effective than topical administration.	[49]
W-INS/*Aureobasidium pullulans*	Branched (1,3;1,6) β-glucan	Wistar rat	An aqueous mixture of β-glucan and polyvinyl alcohol increased the wound contraction ratio by 83% after 11 days, while when treating with cotton gauze, an 85% contraction was observed, but only after 21 days. Additionally, healing time was significantly reduced by 48% using the β-glucan mixture. The accelerating effect of wound healing might be attributed to the release of β-glucan.	[50]
W-S Curdlan, (11.1 mg/mL)/*Alcaligenes faecalis*	Linear (1,3) β-glucan	Diabetic/non-diabetic C57Bl/KsBom mouse	Significantly higher wound closure rates were observed in diabetic mice given topical applications of β-glucan compared to the placebo-treated mice. A more cell-rich and vascularized granulation tissue and an increase in the reepithelialization were observed.	[51]
W-S Betafectin, (2 mg/kg i.v.)/*Saccharomyces cerevisiae*	Branched (1,3;1,6) β-glucan	Fisher-344 rat	Systemic β-glucan treatment resulted in enhanced migration of neutrophils to the site of inflammation and improved antimicrobial function. Polymorphonuclear cells obtained from β-glucan-treated animals showed a heightened respiratory burst and a reduced bacterial load in a pulmonary model of infection.	[52]
Imuneks (100 mg/kg p.o.)/*Saccharomyces cerevisiae*	Branched (1,3;1,6) β-glucan	Sprague-Dawley rat	An oral administration of β-glucan improved impaired anastomotic wound healing in rats treated with corticosteroids over a long period of time. Increased macrophages and fibroblast population were observed in samples from β-d-glucan-treated animals.	[53]

Note: W-INS, water-insoluble; W-S, water-soluble; i.v., intravenous; p.o., per oral.

**Table 3:** Summary of data collection from human clinical studies using β-glucan as a topical remedy in the treatment of ulcers of different etiology.

Type of β-Glucan (Source)/Placebo	Structure of β-Glucan	Ulcer Type	No. of Participants β-Glucan/Placebo	Treatment Duration	Result	Ref.
3% W-INS (*Saccharomyces cerevisiae*)/-	Linear (1,3) β-glucan	venous	12/-	90 d	A 55.2% reduction of the ulcer area was documented at the 90th day. β-glucan increased epithelial hyperplasia, as well as increased inflammatory cells, angiogenesis and fibroblast proliferation.	[54]
2% W-S (*Saccharomyces cerevisiae*)/methylcellulose	Branched (1,3;1,6) β-glucan	diabetic	27/27	12 w	An 87% reduction of the ulcer was documented in the β-glucan group compared to a 56% reduction in the control group. No serious adverse effects in the β-glucan group were recorded.	[55]
W-S (*Saccharomyces cerevisiae*)/-	Branched (1,3;1,6) β-glucan	leg	26/-	12 w	An average wound surface area reduction of 41% was observed; one wound healed; 20 decreased in size; four remained static; and two increased in size.	[56]
W-S (*Saccharomyces cerevisiae*)/-	Branched (1,3;1,6) β-glucan	diabetic	22/-	20 w	The time for complete healing averaged 10.8 weeks (range 6–20 weeks). No adverse effects were recorded during the treatment.	[57]

Note: W-INS, water-insoluble; W-S, water-soluble; d, days; w, weeks.
